# Nurses’ roles, interventions, and implications for management of rheumatic diseases

**DOI:** 10.1007/s00296-024-05603-7

**Published:** 2024-05-02

**Authors:** Dana Auyezkhankyzy, Umida Khojakulova, Marlen Yessirkepov, Ainur B. Qumar, Olena Zimba, Burhan Fatih Kocyigit, Mazlum Serdar Akaltun

**Affiliations:** 1 Department of Emergency Medicine and Nursing, South Kazakhstan Medical Academy, Shymkent, Kazakhstan; 2 Department of Biology and Biochemistry, South Kazakhstan Medical Academy, Shymkent, Kazakhstan; 3 Department of Health Policy and Management, Asfendiyarov Kazakh National Medical University, Almaty, Kazakhstan; 4 Department of Rheumatology, Immunology and Internal Medicine, University Hospital in Krakow, Kraków, Poland; 5 National Institute of Geriatrics, Rheumatology and Rehabilitation, Warsaw, Poland; 6 Department of Internal Medicine N2, Danylo Halytsky Lviv National Medical University, Lviv, Ukraine; 7 Department of Physical Medicine and Rehabilitation, University of Health Sciences, Adana City Research and Training Hospital, Adana, Türkiye; 8 Faculty of Medicine, Department of Physical Medicine and Rehabilitaton, Gaziantep University, Gaziantep, Türkiye

**Keywords:** Rheumatology, Arthritis, Nurses, Nurse specialists, Musculoskeletal diseases

## Abstract

Nurses have become integral members of multidisciplinary teams in managing rheumatic diseases, departing from their traditional patient care roles. This article provides a comprehensive review of nurses’ roles, interventions, and impacts in several rheumatic diseases, including rheumatoid arthritis, osteoarthritis, spondyloarthritis, gout, systemic lupus erythematosus, and polymyalgia rheumatica. It has been demonstrated that care under nursing supervision is effective and safe, with benefits including disease management, quality of life, and treatment adherence. In addition, nurses play a crucial role in promoting health, educating patients, and administering biological disease-modifying anti-rheumatic drugs. The COVID-19 pandemic has highlighted the significance of telehealth services and nurses’ role in delivering remote care. However, nursing education and training challenges persist, particularly in standardization and access to postgraduate education. Efforts to enhance the role of nurses in rheumatology care are necessary to optimize patient outcomes and meet the evolving needs of individuals with rheumatic diseases. Collaboration across healthcare institutions, professional groups, and educational facilities is necessary for promoting the continual growth and advancement of rheumatology nurse practice. By equipping nurses with the requisite knowledge, expertise, and resources to deliver top-notch care, we can enhance the well-being of individuals with rheumatic diseases and foster improved overall health outcomes.

**Supplementary Information:**

The online version contains supplementary material available at 10.1007/s00296-024-05603-7.

## Introduction

Nurses in modern health systems have undergone tremendous evolution, surpassing their basic patient care responsibilities [1]. The growth of nursing roles is especially noticeable in fields including Internal Medicine, Geriatrics, Rheumatology, and Rehabilitation, where nurses significantly impact the development and provision of complete healthcare services. An in-depth investigation is necessary to comprehend the extensive range and profound nature of nurses’ responsibilities in these domains [2–5]. This investigation should encompass the practices followed by institutions and the professional guidelines established by prominent healthcare organizations.

Specialist nurses are registered healthcare professionals who have accumulated experience in a particular area of nursing, obtained further nursing credentials, and have been authorized to work as experts with advanced knowledge in a specific clinical specialty. They have duties for conducting clinical practice, teaching, and consultation [6]. Specialist nurses offer instruction on the significance of nutrition, monitoring, weight management, dietary habits, and consistent use of drugs. They also educate patients on identifying indications of complications and give supportive services to aid patients in self-managing their clinical signs [7].

European League Against Rheumatism (EULAR) encourages rheumatology nurses to engage in close cooperation with the patient (including closest family members), the rheumatologist, and other members of the medical professional team, with a mutual emphasis on healthcare and results [8]. Studies conducted on patients with rheumatoid arthritis (RA) have demonstrated that nurse-led care is widely accepted, similarly efficient, and safe as physician-led care. Additionally, from the patient’s point of view, nurse-led care is convenient and easily reachable [9, 10]. The development and broad adoption of telehealth initiatives have the potential to create new opportunities for rheumatology nurses and improve accessibility [11].

## Aim

This article investigates and illuminates nurses’ multifaceted roles in managing rheumatic disorders. It aims to investigate the evolution of nursing roles in rheumatic disease care, in which nurses play vital parts in patient care beyond their typical tasks. The study aims to assess nurses’ contributions to health promotion and patient education in rheumatology, particularly in promoting self-management and enhancing treatment adherence.

## Search strategy

The relevant papers were acquired from Web of Science, Scopus, Medline/PubMed, Scopus, and DOAJ through a search using the terms “nurses” or “nurse specialists” or “physician-nurse relations” or “nurse-patient relations”, and “rheumatic diseases” or “rheumatology” or “arthritis” or “musculoskeletal diseases”. Only articles written in the English language were considered. No precise timeframe was determined. In addition, we thoroughly examined the references mentioned in the articles retrieved through our search methodology and chose the ones that we deemed pertinent. The search methodology was devised based on the criteria outlined by Gasparyan et al. [12].

## Nurses involvement in the management of rheumatic diseases

### Rheumatoid arthritis

Worldwide, the growing need for rheumatology services has forced the enhanced use of non-physician medical professionals, including rheumatology nurse specialists, to deliver care and support as part of a multidisciplinary team [13]. The current supply of rheumatologists is insufficient to meet the worldwide demand for rheumatology treatments, and this disparity is projected to deteriorate dramatically [14]. One strategy recommended to address the increasing need for rheumatologists is to utilize non-physician professionals, such as specialty nurses, more extensively within nurse-led care management [9].

In a study of active RA patients, participants were randomly assigned to nurse-led or physician-led care groups. Both groups exhibited substantial enhancement in disease activity, and there was no disparity in disease activity. This study demonstrated that nurse-led care is not inferior to traditional approaches for handling RA patients [15]. Dougados et al. [16] showed that the nurse-led approach had beneficial effects on managing comorbidities in RA. This study emphasized the advantages of introducing a program led by nurses to educate patients with RA. This program enhanced the identification and control of simultaneous medical conditions and enabled patients to track the severity of their illness.

Thurah et al. [17] performed a meta-analysis to compare nurse-led follow-up with the traditional strategy in patients with RA. The findings indicated that there was no discernible difference in disease activity after one year between the nurse-led follow-up and the conventional physician-led monitoring. The findings of a meta-analysis indicated that the nurse-led approach was beneficial for managing fatigue [18].

A randomized controlled clinical trial found that nurse-led care resulted in fewer unexpected hospitalizations and extra clinic appointments in RA patients than the usual strategy. Furthermore, the study discovered that the nurse-led strategy resulted in substantially lower overall expenses for RA treatment, laboratory testing, imaging, corticosteroid injections, and daily care visits [19].

### Osteoarthritis

Osteoarthritis (OA) is the most common type of arthritis that is diagnosed on a global scale and is a major contributor to disability and diminished quality of life. Future forecasts indicate a substantial increase in the prevalence of OA as the population ages [20, 21]. Kangeswari et al. [22] assessed the efficacy of a nurse-led intervention in knee OA. The intervention group participants got personalized counseling through the use of cards and presentations in a total of six sessions conducted over six days. Exercise-related sessions were held following all educational counseling meetings. The study indicated that the nurse-led intervention improved the quality of life for patients with knee OA in all subscales. Based on these findings, the researchers conclude that a nurse-led intervention and education, which includes home isometric exercise, enhances the functional level and promotes the quality of life in patients with knee OA. This intervention was self-sufficient, suitable for utilization at home, economically efficient, and simply adjustable for patients with knee OA.

In a randomized controlled study, nurses administered ten sessions of instruction on pain-coping techniques to individuals suffering from chronic pain related to OA, and the control group continued with routine care [23]. The results confirmed the efficacy of pain coping strategies proposed by nurses. Implemented within a practical context, this method had a beneficial effect on pain caused by OA and other symptoms associated with OA.

Kwok et al. [24] examined the efficacy of a 60-minute consultation conducted by a specialized clinical nurse in individuals with hand OA during a rheumatology routine. The findings of this study indicate that a short consultation and phone conversation with a clinical nurse specialist, as a part of regular services, seem to enhance the physical aspect of health-related quality of life in hand OA. The expressed hand pain and disability, as evaluated by a specific indicator of hand function, remained unchanged following the consultation. Following the intervention, there was an observed increase in the utilization of assistive equipment and paracetamol. The majority of participants expressed satisfaction with the education.

Research findings indicate that nurse-led procedures have been shown to benefit individuals with OA. Nevertheless, the absence of standardization among procedures is remarkable. Hence, the establishment of standardized protocols would be more beneficial for OA patients.

### Spondyloarthritis

Spondyloarthritis (SpA) is a comprehensive condition with several subtypes. While each subtype of SpA exhibits distinct clinical characteristics, there are shared clinical signs and comorbidities across all subtypes [25]. Different techniques are currently accessible for evaluating the activity of a disease, and the frequency of measures varies depending on the severity of the condition. In instances of significant disease activity, it is advisable to do more frequently scheduled examinations [26]. Nevertheless, conducting frequent assessments might pose challenges for physicians working in clinics with a large number of patients.

Molto et al. [27] assessed a nurse-led intervention for self-management and self-evaluation of disease severity in individuals with axial SpA (axSpA). The researchers developed an intervention that incorporated self-management techniques, a video tutorial with a series of gradually increasing exercises that can be done at home, and self-evaluation using a video that explains the value of closely tracking disease severity utilizing predefined tools. The nurse instructed the participants in the intervention group on how to obtain, compute, and submit the severity of the disorder. The control group was not provided with information regarding the significance of medical condition monitoring and were not given any educational resources on exercising. However, they were educated about the complications linked to comorbidities at the beginning of the study. While the primary endpoint (coping) did not show any statistical significance, there was substantial evidence favoring this program for the change in disease activity, frequency and length of home exercise sessions in the active group, and physical activity levels.

A randomized controlled clinical trial assessed the efficacy of a nurse-led intervention within a multidisciplinary care setting. The intervention group demonstrated superiority in disease activity, functional status, quality of life levels, and hospitalization compared to the control group [28].

### Gout

Gout is a condition that involves abnormally high levels of uric acid and the buildup of crystals composed of monosodium urate. The prevalence of gout has been progressively rising in recent years [29]. The randomized controlled trial compared the nurse-led program and the standard outpatient follow-up. The program, guided by nurses, was linked to a significant increase in the acceptance of urate-lowering therapy and compliance with the treatment. A higher proportion of patients who received nurse-led care achieved serum urate levels < 360 µmol/L following two years compared to those who received standard care. The provision of gout care led by nurses was cost-effective compared to standard care [30]. Fuller et al. [31] delivered a questionnaire to follow-up gout patients and gathered data on nurse-led care. Subjects who had previously received nurse-led care for gout reported more satisfaction, increased knowledge about the disease, higher likelihood of receiving urate-lowering medicine, and less flare-ups in the preceding year compared to those who had received care led by physicians. Phang et al. [32] developed a telehealth program led by nurses to increase the dosage of urate-lowering treatment. Telemedicine led by nurses proved to be useful and secure for managing gout. The findings validated the advantage of telemedicine in enhancing healthcare accessibility and diminishing healthcare usage. A systematic review revealed that interventions conducted by nurses, such as education and coaching on lifestyle habits, resulted in enhanced compliance with treatment. In general, nurses are essential members of multidisciplinary groups and can significantly impact collaboration on decisions, objective establishment, patient guidance and education assistance, and the provision of suitable contacts [33].

### Other inflammatory rheumatic diseases

A randomized controlled trial in systemic lupus erythematosus (SLE) evaluated the effectiveness of transitional care [34]. The transitional care lasted for 12 weeks. The program included four organized evaluations, associated interventions, and subsequent phone check-ins. Two nurses with a master’s degree and extensive expertise in the nursing field for SLE delivered all interventions. The group receiving transitional care showed considerably larger enhancements in self-care and quality of life compared to the group receiving standard care.

The long-term outcomes of a nurse-led education program in patients with inflammatory arthritis were evaluated. The findings indicated that patients experienced an enhancement in their ability to self-manage their condition during this five-year period; however, there was a slight decline in their physical functionality [35].

Mørk et al. [36] examined the efficacy of a nurse-led prednisolone reduction protocol in comparison to a conventional regimen in polymyalgia rheumatica (PMR) patients. During the first and second-year follow-ups, patients in the nurse-led care group were administered significantly lower dosages of prednisolone. During the second-year follow-up, a greater percentage of patients in the conventional regimen group were still receiving prednisolone treatment. The authors concluded that a rigorous and methodical approach to reducing the dosage of prednisolone was more beneficial than the typical treatment plan.

## Health promotion and nurses in rheumatic and musculoskeletal diseases

Prior studies have demonstrated the beneficial impact of multidisciplinary care on patient outcomes in individuals with rheumatic and musculoskeletal disorders [37, 38]. Individuals with systemic rheumatic diseases have numerous obstacles, such as an uncertain disease trajectory, diverse clinical progression, restricted efficacy of therapy, and variety in symptom manifestation [39]. Nurses play various active roles in rheumatology, including monitoring adverse effects, performing disease-specific tools and questionnaires, providing intravenous drugs, and facilitating self-administration [40]. This could enhance health promotion efforts by alleviating the pressure on healthcare services and mitigating economic expenses. Additionally, it may result in favorable outcomes such as improved patient adherence, enhanced quality of life, and better self-management [41]. Another crucial role of nurses in rheumatic and musculoskeletal disorders is to educate patients and their families. In a systematic review, Wojeck et al. [42] investigated the evidence for nurse-led interventions in patients with autoimmune rheumatic diseases. They concluded that these interventions improved patients’ coping mechanisms, quality of life, and mental health.

In conclusion, nurses can contribute substantially to health promotion by assessing unmet needs in rheumatic and musculoskeletal disorders and identifying appropriate interventions, thereby ensuring the continuity and quality of the chronic care pathway.

### Nurses and biotherapies

Biologic disease-modifying anti-rheumatic drugs (bDMARDs) have made notable progress in treating rheumatic diseases in recent years. Patients receiving bDMARDs may experience both favorable and adverse effects. A heightened susceptibility to infection is a prominent and well-documented negative consequence [43]. Therefore, EULAR recommends influenza and pneumococcal vaccination for patients using bDMARDs [44, 45]. Krasselt et al. [46] found that vaccination rates in RA patients were considerably lower than in the general population. It was thought that misconceptions about bDMARD use being a contraindication for vaccination may be related to these low vaccination rates. Beauvais et al. [47] conducted a multicentre randomized controlled trial to assess the impact of nurse-led education on safety skills in patients receiving bDMARD treatment. This study showed that the education group performed better in infection-related skills than the control group. Additionally, the education group displayed a higher inclination towards vaccination and exhibited better adherence to treatment protocols.

Another critical issue with biologic agents in clinical practice is adherence. Treatment noncompliance and non-persistence are linked to poor clinical outcomes and greater treatment costs [48, 49]. Rheumatology nurses can assist patients with drug adherence. Nurses can improve medication adherence by identifying plausible reasons for patients’ poor adherence, providing reminders as needed, and discussing negative perceptions about treatments [50].

Borras-Blasco et al. [51] administered a 2-hour educational session to patients with RA, psoriatic arthritis, and SpA, transitioning from utilizing prefilled syringes to auto-injector pens for injecting etanercept. Following the educational intervention, the proportion of patients who self-administered etanercept rose from 66 to 94%, whereas the proportion of patients seeking primary care for injection reduced from 23 to 2%. Larsson et al. [52] reported that switching from one of two annual rheumatologist visits to a nurse-led monitoring visit resulted in a considerable reduction in annual expenses for patients with chronic inflammatory arthritis in remission receiving biologic drugs. This can boost patients’ health by improving clinical outcomes while reducing societal costs by lowering disease economic impact. As a result, education delivered by specialized nurses can enhance treatment adherence while also providing significant cost savings for patients utilizing biological agents.

### Nurse functions during the coronavirus disease 2019 (COVID-19) pandemic

The coronavirus disease 2019 (COVID-19) pandemic has not only affected many people around the world but has also severely impacted healthcare. Clinical approaches such as telemedicine were needed throughout the pandemic to prevent transmission and reduce the burden on healthcare systems. While the concept of telehealth services in rheumatology has been a topic of discussion for the past two decades, its utilization has significantly increased due to the COVID-19 pandemic. Several studies in the literature have examined the use of telemedicine in the context of inflammatory rheumatic diseases. In this research, well-planned, customized telerehabilitation regimens were found to have beneficial effects [11, 53]. Although telemedicine experiments during the pandemic time largely engaged physicians, the pandemic experiences revealed that we also need to boost the number and training of specialized rheumatology nurses [54]. In a randomized controlled trial, RA patients with low disease activity or in remission were randomly assigned to patient-reported outcome-based telehealth follow-up by a nurse or rheumatologist or typical outpatient follow-up by a physician. When the patients were evaluated 52 weeks afterward, patient-reported outcome-based telehealth follow-up and traditional outpatient follow-up were found to provide similar disease activity management. The study also found no difference in disease activity between the groups led by nurses and rheumatologists [55].

Telehealth can also be used to improve drug adherence via reminder texts or phone calls, as well as for patient education and counseling [56]. Nurses can conduct telephone or video calls to ensure patients’ active engagement in their care procedures and boost their treatment compliance. In their preliminary investigation, Ozkaraman et al. [57] documented that 37.5% of patients were not provided with information before receiving anti-TNF-α treatment. They also found that telephone nursing consultation did not alleviate the challenges faced by the patients, although it contributed to a partial improvement in treatment adherence. The small sample size is likely responsible for these results [50].

In conclusion, additional rigorous studies are required to expand nurses’ roles in rheumatology by including telehealth. As health systems gain valuable insights from these extraordinary situations, nurses should promptly develop innovative strategies by utilizing health technologies effectively. Telehealth should be integrated into the current curriculum to allow nurses to acquire the necessary expertise to provide secure and proficient healthcare.

### Education of nurses

Establishing a multidisciplinary team in chronic rheumatic diseases is crucial for addressing the diverse requirements of patients, enhancing therapeutic efficacy, and improving treatment adherence. Rheumatology nurses play a crucial role in this multidisciplinary team. Specialized rheumatology nurses are responsible for performing intricate tasks such as evaluating disease activity, administering injections, and monitoring patient progress [58]. Nurses need access to continuing education to develop and maintain their knowledge and skills. However, standardization of education becomes difficult due to the differences in curricula in nursing education in different countries. In some countries, postgraduate education is planned so that rheumatology nurses can take a more active role in the follow-up and treatment of patients. Ryan et al. [59], in their study with nurses who received postgraduate education in rheumatology, reported that receiving postgraduate education increased self-confidence and new clinical and critical evaluation skills. Obtaining postgraduate education in rheumatology poses several challenges. Vlieland et al. [60] demonstrated that the primary obstacles to engaging in postgraduate education are limitations in time and financial resources. While the advantages of specialist rheumatology nurses in terms of clinical and economic gains are recognized, there are issues regarding education. A survey revealed that most rheumatology nurses participated in audits, but less than 25% had presented a poster, and even fewer had published research. The percentage of nurses authorized to write prescriptions is limited to 29%. Less than 20% of nurses received training in administering intra-articular injections or acquired proficiency in performing musculoskeletal ultrasonography [61].

To summarise, the prevalence and standardization of rheumatology nurses’ specialty training appear insufficient for the time being. Nurses should be encouraged to pursue postgraduate and extended education to achieve conformity with national standards and overcome any obstacles they face in doing so.

### EULAR recommendations and perspectives

The current recommendations from EULAR emphasize the importance of rheumatology nurses working with patients, their families, rheumatologists, and other multidisciplinary team members. The goal is to provide comprehensive care and achieve positive outcomes. Patients should have access to a nurse to improve their knowledge and get needs-based education. Patients should have prompt access to a nurse for personalized assistance, which may include telemedicine services. The significance of nurses’ participation in complete disease care to regulate disease activity, mitigate signs, and enhance patient-preferred consequences has been underscored. This strategy is known to result in economical healthcare. Nurses need to address psychological issues and implement procedures to enhance self-management abilities [8].

The volatile characteristics of rheumatic conditions and emerging treatment alternatives occasionally necessitate prompt access to healthcare. Simultaneously, online consultations can facilitate distant care, providing opportunities for novel communication, support, and disease monitoring methods to be utilized [62]. Patients perceive telemedicine presented by nurses and online discussions as providing them with a sense of individualized assistance from a knowledgeable healthcare provider. The service’s quality is comparable to a physician’s typical follow-up to track disease activity [11, 63].

Patients have reported that the care nurses deliver can enhance their quality of life and boost healthcare satisfaction via extended and more comprehensive consultations [64]. Nurse-provided care has the potential to lower hospital admissions and reduce the overall cost of treatment while also improving team collaboration within the health system [65]. Patients highly value the proficiency of nurses, whereas the level of trust patients have in their physicians is the primary factor influencing their assessment of the significance of the care delivered by nurses [66].

**Fig. 1:**
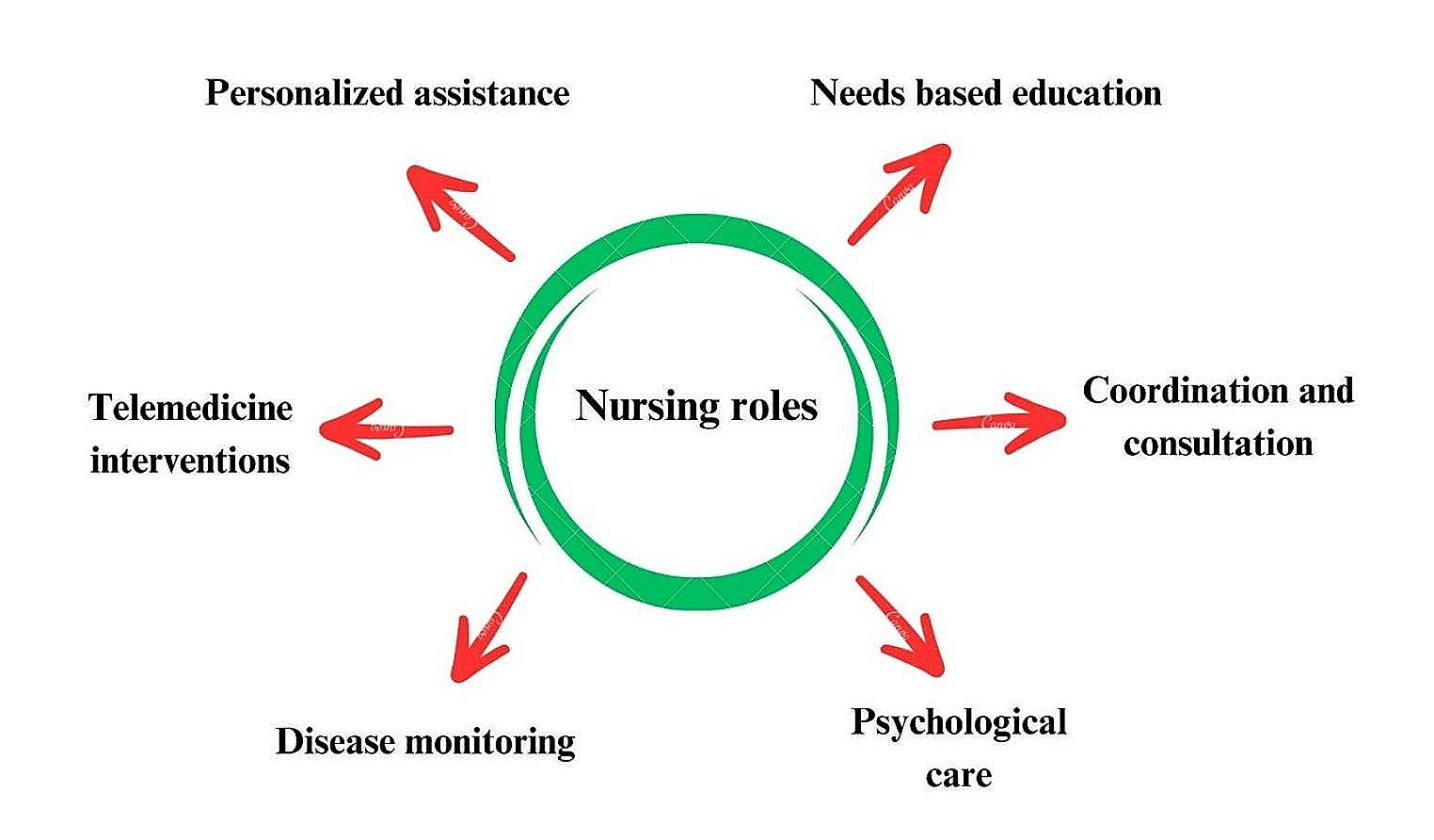
The main roles of specialized nurses

Nurse-led interventions necessitate the self-sufficient execution of disease activity evaluations, health instruction, psychological care, and medical support. Hence, the incorporation of structured education and hands-on training in clinical settings is expected to bolster the responsibilities and skills of rheumatology nurses. Education regarding rheumatic disease handling should encompass theoretical and practical components [67]. The main roles of specialized nurses are visualized in Fig. 1.

Studies have demonstrated that interventions led by nurses in rheumatic diseases have yielded favorable outcomes in terms of treatment, clinical results, and healthcare expenses. However, there is a scarcity of rheumatology nurses in low-middle-income countries, as evidenced in Africa and Middle Eastern countries [13]. Hence, it would be beneficial to formulate innovative strategies and collaborative endeavors for countries under this category.

## Conclusion

Nurses have advanced beyond traditional patient care roles to become essential to multidisciplinary teams, making major contributions to patient education, treatment adherence, and overall health care service. The evidence shows that nurse-led interventions enhance patient outcomes involving disease management, quality of life, and medication adherence across the rheumatic disease spectrum. Furthermore, nurses’ participation in health promotion and bDMARD treatment has been linked to improved patient safety, treatment adherence, and overall clinical results. The COVID-19 pandemic has highlighted the need for telehealth services, with nurses playing a pivotal role in remote care, disease monitoring, and patient support. As healthcare systems advance, it is critical to address nurse education and training issues to maintain consistent, high-quality care delivery across various environments and communities.

### Electronic supplementary material

Below is the link to the electronic supplementary material.

Supplementary Material 1

Supplementary Material 2

Supplementary Material 3

Supplementary Material 4

Supplementary Material 5

Supplementary Material 6

Supplementary Material 7

Supplementary Material 8

Supplementary Material 9

Supplementary Material 10

Supplementary Material 11

Supplementary Material 12

Supplementary Material 13

Supplementary Material 14
